# 1826. Demographics and Risk Factors Associated with HIV Testing among Uninsured Patients Accessing a Free PrEP Program in Northwest Florida

**DOI:** 10.1093/ofid/ofad500.1655

**Published:** 2023-11-27

**Authors:** Colton Boney, Erin G Park

**Affiliations:** The Alabama College of Osteopathic Medicine, dothan, Alabama; Alabama College of Osteopathic Medicine, Gainesville, Florida

## Abstract

**Background:**

Underserved communities in the south are disproportionally affected by the Human Immunodeficiency Virus (HIV). Despite highly effective prevention methods, communities such as sexual minority men (SMM), persons of color (POC) and individuals experiencing multiple social determinants of health (e.g. lack of insurance, poverty) face many obstacles to HIV testing and Pre-Exposure Prophylaxis (PrEP). This study describes demographic characteristics and risk factors associated with HIV testing within the context of a no-cost PrEP program for uninsured persons living in Escambia County, FL.

**Methods:**

Enrollment data from the program was used to characterize the relationship between HIV testing, race/ethnicity, prior PrEP use, among other factors. Chi-square and Fisher Exact tests between HIV testing and risk factors were examined, and program demographics were compared to the county’s census data to see if the program reached more POC. With approval from the Florida Department of Health, OASIS provided a limited data set containing all HIPAA compliant information in their enrollment and HIV test forms. Statistical analysis was conducted using Stata v16.

**Results:**

Of the 107 participants, 13% reported never been tested for HIV and 63.5% reported never been on PrEP. Findings suggest that HIV testing differed by gender, ethnicity, sexual orientation, number of sexual partners, prior STI diagnosis, and sex with an anonymous partner (p< 0.05). For example, White patients were more likely to have never had an HIV test as compared to Black participants (p< 0.05). When reviewed, the program's profile contains 5.53% more people of color as compared to Escambia County's Census demographics for 2020.

**Conclusion:**

Our findings suggest that many factors are associated with HIV testing uptake in this population of uninsured patients served by the OASIS program. Understanding the demographic and health factors associated with lower uptake can help clinicians target populations who can benefit from HIV education and testing.

**Disclosures:**

**All Authors**: No reported disclosures

